# Recovery of dyes and salts from highly concentrated (dye and salt) mixed water using nano-filtration ceramic membranes

**DOI:** 10.1016/j.heliyon.2022.e11543

**Published:** 2022-11-09

**Authors:** Shekh Md. Mamun Kabir, Hassan Mahmud, Harald Schӧenberger

**Affiliations:** a Department of Wet Process Engineering, Bangladesh University of Textiles, Tejgaon, Dhaka, Bangladesh; b Department of Environmental Science & Management, North South University, Dhaka, Bangladesh; c Institute for Sanitary Engineering, Water Quality and Solid Waste Management, University of Stuttgart, Germany

**Keywords:** Nanofiltration (NF) ceramic membranes, Reactive dyes, Salts, Textile wastewater, Separation

## Abstract

In this study, a higher concentration of (reactive dyestuff and salt) mixed water was used to verify the feasibility of separation by membrane techniques. The commercial nano filtration ceramic membrane (MWCO 200 Da) has been used in cross flow mode for separation of dyes and salts from highly concentrated mixed water solution. NF ceramic membrane presents good permeability (pure water flux 54.15 Lm^−2^ h^−1^, TMP 8 bar), 8% dye rejection and reduced salt rejection of NaCl (<8%) and Na_2_SO_4_ (<25%). Consequently, the operation parameters (TMP, temperature) and solution environment (solution pH, salt concentration and dye concentration) have been intensively evaluated for separation efficiency in the NF ceramic membrane process. Significantly, the NF ceramic membrane has performed less rejection to chloride ions than sulphate ions due to the Donnan effect. Solution pH, concentration of salt and dye concentration have shown significant effects on ceramic membrane separation performance. In addition, pollutant removals were achieved with noteworthy values for the chemical oxygen demand for permeate solution also color difference between concentrate and permeate. In conclusion, the strong rejection of dyes by the NF ceramic membranes proves that it can be suitable alternatives for textile wastewater treatment process.

## 1 Introduction

Reactive dyes are mostly popular anionic dyestuff used in textile industry for coloration, especially for cotton dyeing processes [1, 2]. In addition, reactive dyes can form covalent bonds with primary and secondary hydroxyl groups of cellulose; it also can be hydrolyzed in water. Salt and soda addition in reactive dyeing has been widely used for dye exhaustion and fixations on cotton fabric therefore minimize the hydrolysis rate of dyes [3, 4, 5]. Large amount of effluent treatment plants (ETP) is a result of dye with different salts, which causes high chemical oxygen demand (COD), heavy colors and toxic substances [6, 7, 8]. Dyeing effluent prompts not only environmental degradation but also hinder the biological cycles [9, 10, 11]. Moreover, the presence of salts like chlorides, sulphates, heavy metals and unfixed dyes containing azo groups in wastewater are considered as carcinogenic and mutagenic [12, 13, 14]. Therefore, treatment of dye effluent and reuse of dyes, salts and chemicals is a major challenge also paying great interest because of water scarcity and environmental legislation [15]. Traditional effluent treatment process including anaerobic/aerobic biological processes, adsorption, photo catalytic and ozonation has been used for long time [16, 17, 18]; however, most textile dyes have complex aromatic structures and are stable in light and oxidizing agents so it is quite difficult to recovery and reuse of valuable dyes and salts from wastewater [19].

Membrane based separation techniques have been widely used for wastewater treatment because of its adsorption, sieving and electrostatic behaviour [20, 21]. Membrane filtration has been developed by different mechanisms such as microfiltration (pore size between 1000 nm and 100 nm) [22, 23], ultra-filtration (pore size between 100 nm and 2 nm) [24], nanofiltration (pore size below 2 nm) [25] and reverse osmosis (pore size around 0.1 nm) [26]. Microfiltration (MF) is used for removing the suspended matter; on the other hand, ultra filtration (UF) allows the removal of particles and macromolecules [27] but does not remove low molecular weight and water-soluble dyes such as acid, reactive, basic dyes etc [28]. However, nanofiltration (NF) is an effective method for the separation of low molecular weight compounds like divalent salts, different mineral salts and chemicals [29]. The pore size of the nanofiltration membrane from 0.5 to 2.0 nm has shown advantageous effects for high flux, low osmotic pressure and strong rejections towards water-soluble dyes [30]. However, membrane technology has some limitations, such as membrane fouling during operation time, which leads to economic losses [31]. Several researches have been carried out about nanofiltration membranes for the separation of dyes and inorganic salts [32, 33, 34, 35, 36, 37, 38] from textile wastewater and reported that almost 90% dyes were recovered and salt rejection rates for NaCl (<10%) and Na_2_SO_4_ (<30%) were obtained at pressure 1.0–3.0 bar [39]. In general, most of the researchers evaluate the membrane performance by using minimum amounts of dyestuff (0.5 g/L) and salt (1.0 g/L) [39, 40]. However, textile coloration industry uses huge amounts of salts for cotton dyeing with different reactive dyeing mechanism such as cold pad batch, pad jig at (40–60 °C), exhaust method at (80–90 °C) and pad steam at (100–105 °C) [41, 42]. Salt addition in reactive dyeing causes an electrical positive double layer, which reduces the negative electrostatic charge (Donnan potential) on the cellulose surface, which allowing better interaction of Van der Waals force and improves the substantivity [43]. Salt use in reactive dyeing depends on the shade percentage on the fabric, such as for light shade 37 g/L, medium shade 60 g/L and deep shade 80 g/L is used [3]. However, so far there is no research carried out regarding the actual amount of dyestuff and salt (40–60 g/L) used in dyeing industry for verifying the feasibility of separation and reuse by membrane techniques.

Polymer based membranes are mostly used because of the hydrophilic nature of polysulfone and polyethersulfone. The major disadvantages for polymeric membranes are not only instability in organic solvents and at high temperatures but also impairments by alkaline and acidic solutions. Therefore, the utilization of polymeric membrane for separation of textile waste water is quite difficult [27]. Ceramic Nano filtration membrane has been developed in 2000, with a MWCO of 450 Da [44]. Ceramic membranes have shown more advantageous effects such as high chemical, mechanical and thermal stability than polymeric membranes. Although, ceramic membranes are quite expensive, however reimburse by higher fluxes and extended life time [45]. An asymmetric structure of ceramic membrane comprised with a coarse porous ceramic support can be produced by different techniques such as dry-pressing, paste processing or colloidal processing and sol-gel techniques [46]. The brand “inopor-nano” for small and medium scaled nano filtration membrane (MWCO 200 Da) was developed in Germany during the research project “nanomembrane” from 2010 to 2013 [47]. Ceramic membranes are widely used for the separation of water in oil/tar sand mining, potash mining and textile dyeing effluents because of efficient recycling processes including heat recovery due to their resistance against organics and oil residuals and their temperature stability [47, 48]. In the past, most researches about ceramic membrane have been carried out by focusing on the influence of cross flow velocity (CVF), molecular weight cut-off (MWCO) and feed pH of the concentration [[49], [50], [51], [52], [53]]. Nevertheless, limited research has been found in the literature involving NF ceramic membranes use for separation of textile dyeing effluent.

The aim of this study was to investigate and verifying the feasibility of separation of reactive dyes and salts from textile waste water at higher salt and dye concentrations (used in industries) by using NF ceramic membrane consisting of a TiO_2_/ZrO_2_ skin layer. To evaluate an actual condition for reactive dyeing on cotton fabric was set for performance evaluation of nano filtration ceramic membrane was studied.

## 2 Experimental

### 2.1 Membranes

The Ti(Zr)O_2_–NF ceramic membranes LC1SG 2548-B (L8-92.1) were supplied by inopor®, Rauschert Distribution GmbH, Scheßlitz, Germany. The monochannel tubular membrane has a length of 0.5 m with an outer diameter of 10.0 mm and lumen side diameter of 7.0 mm. The effective filtration area of the membrane is 0.012 m^2^. The separation layer is composed of Ti(Zr)O_2_, middle layer materials are ZrO_2_, the intermediate layer materials are TiO_2_ and the support layer is composed of Al_2_O_3_. The surface morphology of the NF ceramic membrane was taken from report “nanomembrane”. Pure water permeability of the NF ceramic membrane has been measured at 40 °C with trans membrane pressure for 8 bar and flow rate 9.3 L/min, the flux obtained accordingly 54.15 L-m^−2^ h^−1^. The MWCO distribution curve for NF ceramic membrane was supplied by the projects “nanomembrane”. The MWCO of the Nano-filtered ceramic membrane arriving at R = 90% pointed at a PEG of molecular weight about 200 Da [FKZ: 03X0080 and KerWas, FKZ: 03XP0096].

### 2.2 Chemicals

**Scheme 1:**
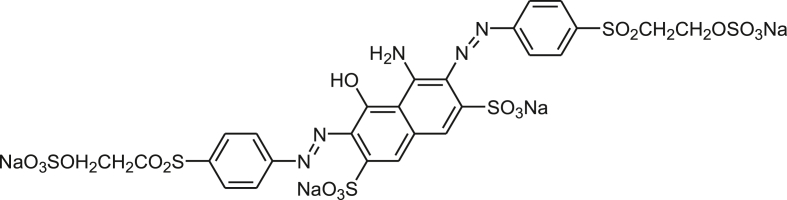
C.I. Reactive Black 5.

In this study, pure water was used which obtained from Fa. Heymann Destilliertes Wasser, Lichtenau, Germany. All the chemicals and salts like NaCl, Na_2_SO_4_ and NaOH (99% purity) were provided by Carl Roth GmbH Co. KG, Karlsruhe, Germany. Mostly used anionic reactive dyes Remazol Deep Black N150 (C.I. Reactive Black 5) (Dystar) (M. W. 991.82 g/mol and λ_max_ 598 nm, Scheme 1) were obtained from Sächsisches Textilforschungsinstitut (STFI), Chemnitz, Germany. C.I. Reactive Black 5 was used without purification prior to use.

### 2.3 Membrane unit

**Figure 1:**
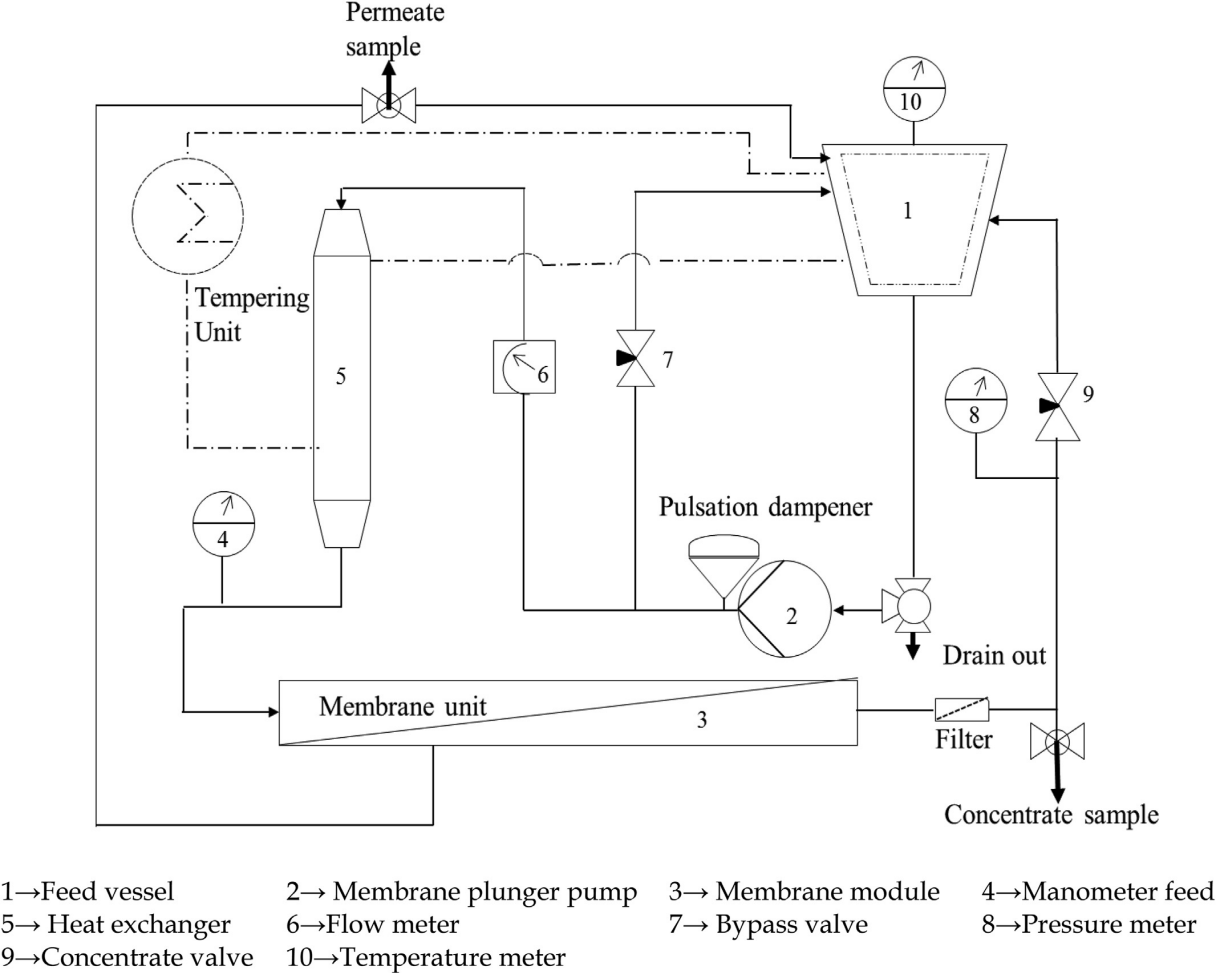
Schematic view of cross-flow membrane plant.

Experiments with the nano filtration ceramic membrane were carried out in a Cross-Flow-Membrane Plant InoMini 2019-07 supplied by Rauschert Distribution GmbH, BU inopor, Scheßlitz, Germany. The feed vessel had a volume of 5 L. Volume flow rate range of the system was 9.3–2.4 l/min, leading to a maximum cross flow velocity of 4 m/s for the monochannel membrane diameter 7 mm. Feed temperature could be controlled by the heat exchanger, which was connected to an additional tempering unit to maintain the exact temperature across the test set-up. The feed was circulated across the membrane by a membrane plunger pump. The membranes were housed in a horizontally fixed monochannel module (manufactured by Andreas Junghans, Frankenberg, Germany). The feed is pumped axially through the tubular ceramic membrane. In the membrane module the feed is divided into permeate and concentrate. By slowly closing the needle valve behind the membrane module, the system was pressurized. Both the concentrate and permeate were recirculated into the feed vessel. The volume flow over the membrane was controlled by a bypass needle valve and a flow meter shown in Figure 1.

This study was carried out with different operating parameters according to the capacity of membrane load shown in Table 1. Reactive dyes in the cotton dyeing usually 60–70% hydrolysed after the dyeing process. Therefore, in this experiment the simulated high-salinity dye liquors were stored for 1 h after mixing with pure water, reactive dye and salt. The duration of each test was more than 30 min with a constant cross flow velocity of 4 m/s at 40–60 °C. In all the experiments, permeate flux was monitored at regular intervals and solutions were collected in triplicate from both concentrate and permeate streams for analysis. pH of the solution was adjusted with sodium hydroxide. After every experiment the membrane was washed and rinsed repeatedly with distilled water and cleaned with sodium hydroxide solution at pH 10–11. After that, the membrane was rinsed with deionised water until the pH turned neutral and pure water flux of the membrane was tested again. Pure water flux of the ceramic membrane must recover to 95% of the initial flux were practiced.

**Table 1:** Operating parameters for testing the nano filtered ceramic membranes.

Transmembrane pressure TMP (bar g)	Temperature (^o^C)	pH	Salt concentration (g/L)	Dye concentration (g/L)	Flow rate (L/min)
8	40	8	40	0.5	9.3
12	50	10	50	1	
16	60	12	60	2	

### 2.4 Analytical methods

The concentration of monosalt NaCl and Na_2_SO_4_ contained in pure water and in dye solution was determined with a conductivity meter (Greisinger, G-1400 Series, GHM Messtechnik GmbH, Gemrany). Solution pH was measured with a digital pH meter (Sensortechnik Meinsberg GmbH, Germany) and pH strips (MACHEREY_NAGEL GmbH, Germany). The concentration of dyes in solution was determined with a UV/Vis spectrophotometer *NANOCOLOR® VIS II* (*MACHEREY-NAGEL* GmbH & Co. KG, Germany). Permeate flux J was determined by the following Eq. (1)

(1) $$J=\frac{V}{\;A.t\;}$$

where V (L) is the volume of permeated water, A (m^2^) is the active surface area of the membrane and t (h) is the permeation duration.

Salt and dye rejection was calculated by the following Eq. (2)

$$Rejection\;\%=\frac{Concentartions\;of\;solutes\;in\;concentrate\;\left(\frac{g}{l}\right)-Concentrations\;of\;solutes\;in\;permeate\left(\frac{g}{l}\right)\;}{Concentrations\;of\;solutes\;in\;concentrate\;\left(\frac{g}{l}\right)}$$

(2) $$R\left(\%\right)=\left(1-\frac{C_{p}}{C_{f}}\right)\times 100\;\%$$

where C_p_ (g/L) and C_f_ (g/L) are the concentrations of solutes in permeate and concentrate respectively.

The color difference (ΔE) values of the concentrate and permeate solution were measured using UV/Vis spectrophotometer. The Color differences, i.e., CIE Lab color deviation (ΔE) was calculated by the following Eq. (3)

(3) $$\Delta E=\sqrt{{\left({\Delta L}^{*}\right)}^{2}+{\left({\Delta a}^{*}\right)}^{2}+{\left({\Delta b}^{*}\right)}^{2}}$$

The values of L∗, a∗ and b∗ for a given color locate its position in the three-dimensional CIELab color space where, ΔL∗ = deviation of lightness (L_concentrate_ − L_permeate)_, Δa∗ = deviation of color in green-red axis (a_concentrate_ − a_permeate_) and Δb∗ = deviation of color in yellow-blue axis (b_concentrate_ − b_permeate_) were evaluated according to the AATCC test method 173–2006 under D_65_. The COD (Chemical oxygen demand) and TOC (Total Organic Carbon) (TOC-L, Shimadzu, Japan) for concentrate and permeate sample was estimated by ISO 15705 and DIN EN 1484 test method accordingly.

## 3 Results and discussion

### 3.1 Effect of transmembrane pressure

**Figure 2:**
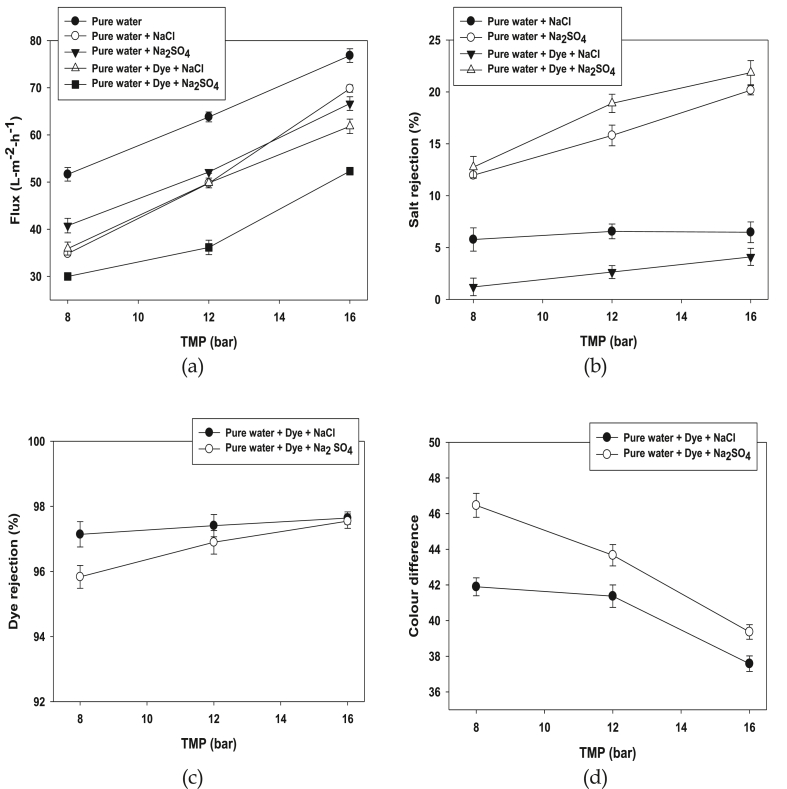
Permeate flux (a), Salt rejection (b), Dye rejection (c) and Colour difference (d) of the nano-filtration ceramic membrane LC1 SG 2548-B (L8-92.1) at different transmembrane pressures using dye and different salts NaCl and Na_2_SO_4_ (Conditions: Dye conc: 0.5 g/L, Salt conc: 40 g/L, pH: 8, cross flow velocity (CFV): 4 m/s and Temp = 40 °C).

The general driving force behind permeation rates of nanofiltration ceramic membrane is the transmembrane pressure (TMP). As ceramic membranes show no swelling or compaction effects, an almost linearly permeate flux increase with TMP was expected and it is shown in Figure 2 (a) that permeate flux increases linearly with operating pressure. The resistance could be strengthened by concentration polarization at higher transmembrane pressure [54]. From Figure 2 (a), it is clear that the permeate flux values of pure water shows a linear increase, however flux decreased when added with different salt and dyestuff. In addition, the biggest drop observed dye with Na_2_SO_4_, it can be explained that large inorganic ions create high charge density, which reduced the permeation flux. On the other hand, salt rejection percentage linearly increases with increasing pressure as shown in Figure 2(b). On the other hand, salt rejection percentage linearly increases with increasing pressure as shown in Figure 2(b). The results discussed that the membrane rejection to chloride ions increases from 0.65 to 4.9% for dye with NaCl, 4.6–7.49% for pure water with NaCl and rejections of sulphate ions from 11.8 to 20.6% for pure water with Na_2_SO_4_, 11.57–22.85% for dye with Na_2_SO_4_. The less rejection of NaCl also mentioned in many studies [55], it can be explained that Donnan effect plays a significant role for separation of various charged species through the porous channel of the membranes [56]. Dye rejection by NF ceramic membrane is displayed in Figure 2(c), it can be seen that dye rejection remains almost stable from 96.75 to 97.8% for dye with NaCl and from 95.5 to 97.8% for dye with Na_2_SO_4_ in a TMP range of 8–16 bar. This could be attributed to the highest rejection rate of nano-filtration membrane surface, because of higher electrostatic repulsion between dye molecules and membrane [54]. Color difference (ΔE) is a term used to describe the color removal efficiency by the nanofiltration membrane from concentrate to permeate. Higher color difference represents better color separation efficiency. Dye with Na_2_SO_4_ has shown higher color difference than dye with NaCl in Figure 2(d). It can be explained that small inorganic ions is mainly convective (pressure dependent) because they can enter the pores in the membrane easily, on the other hand large inorganic ions creates high charge density so the transport is mainly diffusive. In addition, when the pressure increases the color difference reduces, because more pressure influences more color permeates.

### 3.2 Effect of temperature

**Figure 3:**
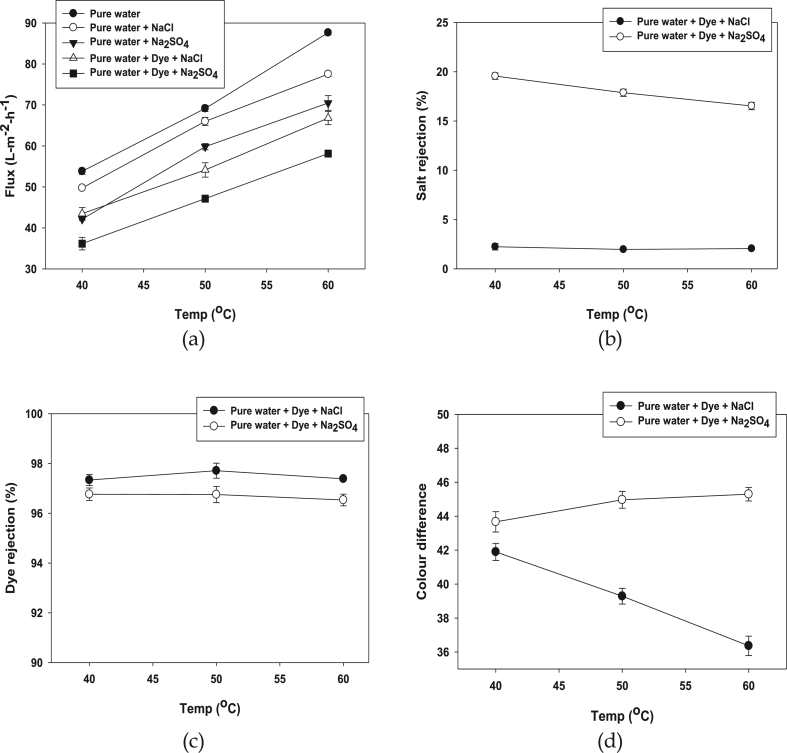
Permeation flux (a), Salt rejection (b), Dye rejection (c) and Colour difference (d) of the NF ceramic membrane at different temperature using dye and different salt NaCl and Na_2_SO_4_ (Conditions: Dye conc: 0.5 g/L, Salt conc: 40 g/L, pH: 8, CFV: 4 m/s and TMP = 12 bar).

As can be seen from Figure 3 that, permeation flux and dye/salt rejection by NF ceramic membrane at 40–60 °C. It is clearly shows that permeation flux goes up with the increased temperatures. The flux at 60 °C (87.98 L. m^−2^ h^−1^) for pure water is about 1.6 times higher than that 40 °C (52.99 L. m^−2^ h^−1^). This can be explained by higher temperatures, the resistance of total mass transfer is reduced at so the viscosity decreases and diffusion coefficient increases in Figure 3a [57]. Figure 3b shows that salt rejection rate decreases when the temperature increases. It is apparent that salt rejection of dye with Na_2_SO_4_ is 19.89% at 40 °C and 16.10% at 60 °C; on the other hand, the rejection of dye with NaCl is 2.59% at 40 °C and 1.98% at 60 °C. The salt rejection percentage is quite low because salt solutes distribute more evenly between the solution and the membrane phases at high temperature resulting in lower rejection [58]. An insignificant (less than 1%) thermal effect from temperature at 40–60 °C has shown in Figure 3c, 98.03% dye rejection was found for dye with NaCl on the other hand 97.07% dye rejection was obtained for dye with Na_2_SO_4_. The colour difference of dye with NaCl and dye with Na_2_ SO_4_ in different temperature is shown in Figure 3d. When the temperature increases the colour difference of dye with NaCl reduced linearly, on the other hand, insignificant color difference obtained by dye with Na_2_SO_4_. This result can be explained that salt solutes distribute between the solution and the NF membrane.

### 3.3 Effect of solution pH

**Figure 4:**
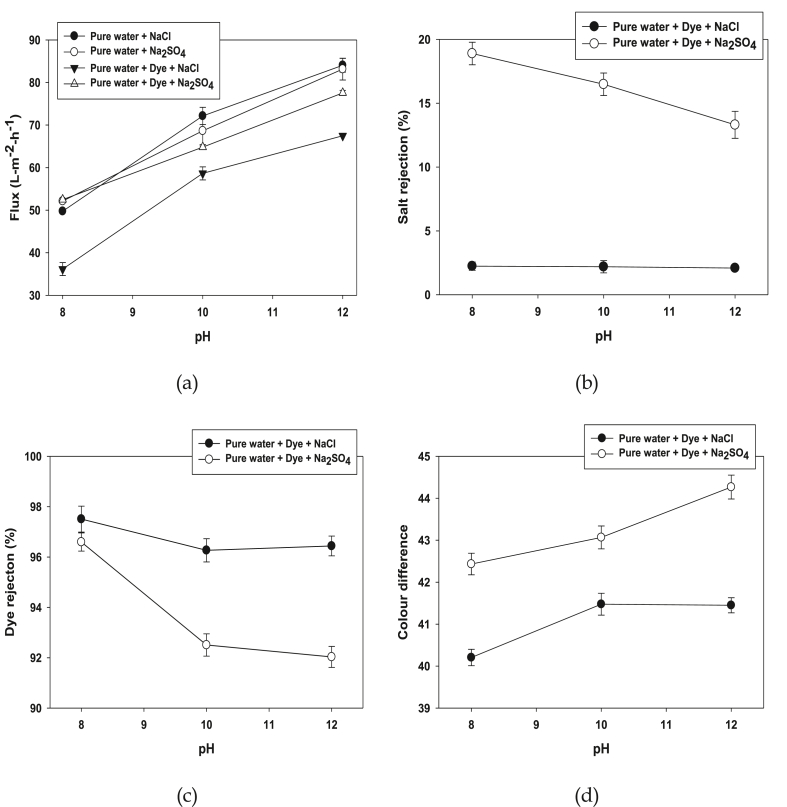
Permeation flux (a), Salt rejection (b), Dye rejection (c) and Colour difference (d) of the NF ceramic membrane at different pH using dye and different salt NaCl and Na_2_SO_4_ (Conditions: Dye conc: 0.5 g/L, Salt conc: 40 g/L, CFV: 4 m/s, TMP = 12 bar and Temp: 40 °C).

The separation efficiency of the ceramic membrane process is mainly influenced by solution pH because of zeta potential, isoelectric point and point of zero charge properties [59]. The NF ceramic membrane surface charge sites for protonation and deprotonation depends on the magnitude and polarity of charges at various pH values. It is clearly shown in Figure 4a permeation flux increased when the pH increased from 8 to 12. It can be noted that the permeation flux of dye solutions containing NaCl and Na_2_SO_4_ reduced 1.25% than pure water with NaCl and Na_2_SO_4_ because in higher pH conditions dye may aggregates, which enhanced the rate of membrane fouling. Salt rejection percentage with different pH presented in Figure 4b as sulphate ion rejection rate has shown 19.55% at pH 8 and then declines to 13.76% at pH 12. On the other hand, insignificant rejection 2.7% of chloride was obtained at different pH because ionic nature of different salt solutions. In general, rejection of dye influence by different pH (8–12) has represented in Figure 4c. Dye with NaCl and dye with Na_2_SO_4_ has shown almost 97–98% dye rejection at pH 8, however 1% dye rejection reduced when pH increased at 12. It can be explained that higher alkaline conditions dye may aggregates, which affects the membrane surfaces and dye rejection reduced. Colour difference of concentrate and permeate solutions also influenced by different pH is shown in Figure 4d. Dye with Na_2_SO_4_ has shown higher color difference than dye with NaCl, when the pH increased, colour difference also increased because intensified charge density of dye with different salt leads to higher colour rejection from concentrate to permeate.

### 3.4 Effect of salt concentration

**Figure 5:**
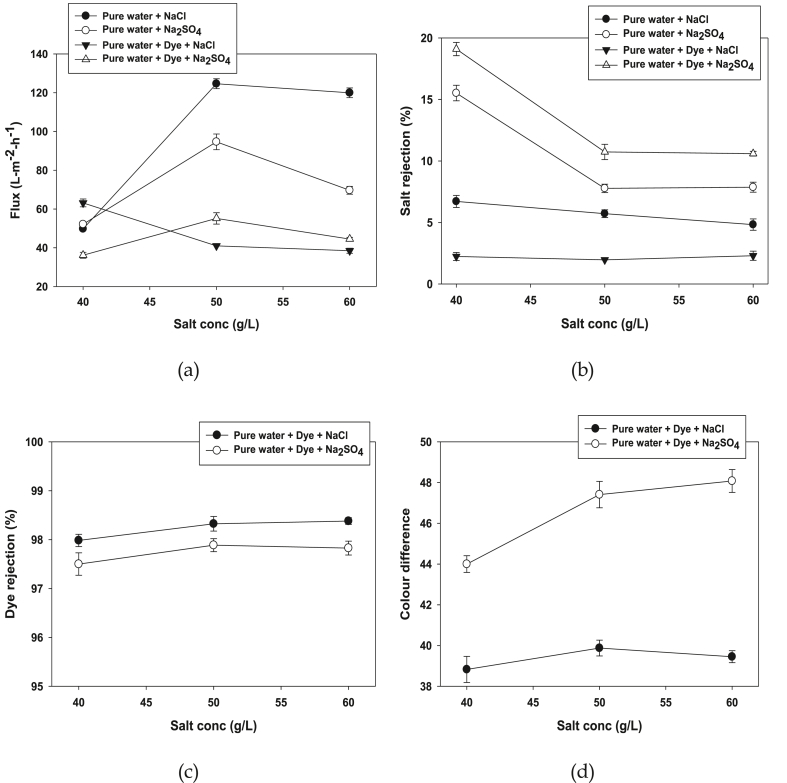
Permeation flux (a), Salt rejection (b), Dye rejection (c) and Colour difference (d) of the NF ceramic membrane at different salt concentration (40–60 g/L) using dye and different salt NaCl and Na_2_SO_4_(Conditions: Dye conc: 0.5 g/L, pH: 8, CFV: 4 m/s, TMP = 12 bar and Temp = 40 °C).

Effect of salt concentration on the permeation flux is illustrated in Figure 5a. Except for dye with NaCl, the flux increased with salt concentration was increased from 40 to 50 g/L. Permeation flux apparently decreases for all studied fluid systems were observed. It can be explained that dye aggregations may occurred in higher salt concentrations, so shielding effects formed on the membrane surface. The flux decline (1.69–1.96%) obtained dye with different salt concentration than pure water with salt. Salt rejection as a function NF of ceramic membrane performance, salt rejection dependency from pure water and salt was studied, which is shown in Figure 5b. The ceramic membrane showed a reduced rejection to the inorganic anions with increased concentration of salt. Rejection of sulphate ions dropped obtained from 19.59 to 10.59% for dye with Na_2_SO_4_ and found 15.96 to 7.38% for pure water with Na_2_SO_4_ when the salt concentration increases from 40 to 60 g/L. On the other hand, rejection of chloride ions drops from 7.24 to 4.31% for pure water with NaCl and 2.73 to 1.95% for dye with NaCl, when the salt concentration increases. The higher sulphate ions retained by the ceramic membrane than chloride ions might be due to polarization effect [60, 61], charge density and diffusion coefficients [62] of the inorganic ions. Dye rejection percentage with the presence of different salt concentration has been depicted in Figure 5c.

Dye rejection is found 98% while using dye with NaCl and insignificant change was observed when the salt concentration increased. Dye rejection is 97.50% with using different salt concentrations. The colour difference with different salt concentrations shows in Figure 5d. The colour difference of dye with Na_2_SO_4_ and dye with NaCl increased with increasing salt concentration. This is caused by the salt concentrations weekend the Donnan effect. In addition, more salt ions make homogenous dispersion of dye particles in water and may help to minimize the blockage of the membrane surface so the colour difference increased.

### 3.5 Effect of dye concentration

**Figure 6:**
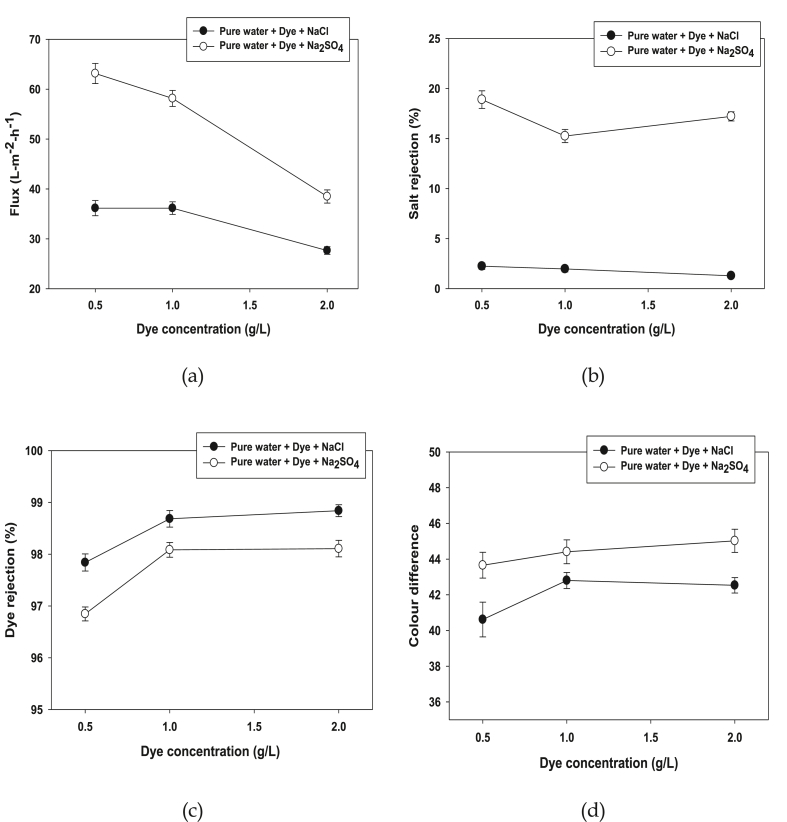
Permeation flux (a), Salt rejection (b), Dye rejection (c) and Color difference (d) of the nano-filtered ceramic membrane at different dye concentration (0.5–2 g/L) and different salt NaCl and Na_2_SO_4_ (Conditions: Salt conc: 40 g/L, CFV: 4 m/s, TMP = 12 bar and Temp: 40 °C).

The impact of dye concentration on the membrane separation process has been investigated by changing the dye concentration from 0.5 to 2.0 g/L as shown in Figure 6. Permeation flux declined when the dye concentration increased from 0.5 to 2 g/L shown in Figure 6a. It is clearly shown that 1.67% flux drop for dye with Na_2_SO_4_ and 1.32% flux drop for dye with NaCl has obtained. This can be caused by dye aggregation increased when the dye concentration increased which results into a cake layer on the membrane surface. Salt rejection of different dye concentration has been analyzed and the results are depicted in Figure 6b. Rejection of sulphate ions drops from 19.55 to 16.77% when the dye concentration increased from 0.5 to 2.0 g/L. Furthermore, chloride ion drops from 2.59 to 1.27%. Salt rejection deceased because of reduced permeation flux. It reveals that the dye concentration in dye solution has an effective impact on the NF ceramic membrane performance as shown in Figure 6c. It is clearly shown that; 97% and 97.85% dye rejection obtained at 0.5 g/L dye with Na_2_SO_4_ and dye with NaCl accordingly. When the dye concentration slightly increased to 2.0 g/L dye rejection rate increased to 98.15 and 98.8% respectively. So, dye rejection rate increased when the dye concentration increased. Insignificant color difference obtained when the dye concentration increased as shown in Figure 6d. This can be explained by the fact, that more dye concentration increases dye aggregation which can be affected the concentrate and permeate colour of the sample. The influence of dye concentration for NaCl and Na_2_SO_4_ by the NF ceramic membrane separation regarding to the chemical oxygen demand (COD) and TOC given in Table 2. The COD for dye with NaCl shows almost ten time lower from concentrate to permeate. However, it is noticeable that COD values significantly decrease from concentrate to permeate, in case of dye with Na_2_SO_4_. NF ceramic membrane is very efficient techniques for adaptation and separation of various types of pollutants like organic, mineral and heavy metals form solutions. In addition, the separation efficiency of NF ceramic membrane shows higher rates for dye with Na_2_SO_4_ than dye with NaCl. This can be explained the polarization effects enhance the removal of pollutants. In contrast, a significant TOC level has observed from concentrate to permeate, both for dye with two salts. Colour could be greatly removed by the NF ceramic membrane treatment. It can be explained that the sulphate ions being more effectively retained by the ceramic membrane than chloride ions because of different ionic charge density. The treatment of the dye solution is satisfactory with a high level of colour and organic pollution abatements by NF ceramic membrane.

**Table 2:** Influence of NF ceramic membrane separation by different dye concentration.

Salt	Sample		Dye Concentration		
			0.5 g/L	1 g/L	2 g/L
NaCl	Concentrate	Solution			
		COD	584 mg/L	591 mg/L	600 mg/L
		TOC	141 mg/L	172 mg/L	346 mg/L
	Permeate	Solution			
		COD	58.6 mg/L	60.7 mg/L	61 mg/L
		TOC	6.79 mg/L	16.4 mg/L	35.7 mg/L
Na_2_SO_4_	Concentrate	Solution			
		COD	519 mg/L	624 mg/L	625 mg/L
		TOC	93 mg/L	171 mg/L	408 mg/L
	Permeate	Solution			
		COD	1.15 mg/L	1.26 mg/L	1.9 mg/L
		TOC	13.1 mg/L	14.7 mg/L	72.4 mg/L

## 4 Conclusion

The investigation of ceramic nanofiltration membranes in synthetic textile waste water showed up to 98% of dye rejections and a removal of Na_2_SO_4_ salts at 22.85% and NaCl salts at 7.4% at pressures up to 16 bars. High fluxes achieved by adjusting operating parameters like transmembrane pressure, temperature, salt concentration, pH and dye concentration have proven the general cleaning efficiency of NF ceramic membrane for textile wastewater. Higher transmembrane pressures enhanced the salt rejection percentage. However, the dye rejection was nearly hundred percent. On the other hand, dye and salt rejection did not affected by higher temperature. By increasing the temperature, colour difference of dye with NaCl reduced in linear state, on the other hand, insignificant color difference obtained by dye with Na_2_SO_4_. In higher alkaline conditions, dye may aggregate, which affect the membrane surfaces so dye and salts rejection is reduced. Sulphate ion rejection obtained 19.55% at pH 8 and declined 13.76% at pH 12. On the other hand, insignificant rejection obtained for dye with NaCl. With increasing salt concentration in the feed solution, the rejection of salts decreased due to the shielding of the electrical double layer influencing the electric repulsion. When the salt concentration increases salt rejection rate decreased almost 2% also dye rejection rate not influenced by salt concentration. With increasing, the dye concentration permeation flux declined because of dye aggregation on the membrane surface. Insignificant salt and dye rejection rate obtained with increasing the dye concentration.

## Declarations

### Author contribution statement

Shekh Md. Mamun Kabir: Conceived and designed the experiments; Performed the experiments; Analyzed and interpreted the data; Wrote the paper.

Hassan Mahmud: Analyzed and interpreted the data.

Harald Schoenberger: Analyzed and interpreted the data; Contributed reagents, materials, analysis tools or data.

### Funding statement

This work was supported by Bangladesh University of Textiles.

### Data availability statement

Data included in article/supp. material/referenced in article.

### Declaration of interest’s statement

The authors declare no conflict of interest.

### Additional information

No additional information is available for this paper.
